# Characterization of Pectinase from* Bacillus subtilis* Strain Btk 27 and Its Potential Application in Removal of Mucilage from Coffee Beans

**DOI:** 10.1155/2017/7686904

**Published:** 2017-09-11

**Authors:** Oliyad Jeilu Oumer, Dawit Abate

**Affiliations:** ^1^Department of Biology, Ambo University, P.O. Box 19, Ambo, Ethiopia; ^2^College of Natural Science, Addis Ababa University, P.O. Box 1176, Addis Ababa, Ethiopia

## Abstract

The demand for enzymes in the global market is projected to rise at a fast pace in recent years. There has been a great increase in industrial applications of pectinase owing to their significant biotechnological uses. For applying enzymes at industrial scale primary it is important to know the features of the enzyme. Thus, this study was undertaken with aims of characterizing the pectinase enzyme from* Bacillus subtilis strain Btk27* and proving its potential application in demucilisation of coffee. In this study, the maximum pectinase activity was achieved at pH 7.5 and 50°C. Also, the enzyme activity was found stimulated with Mg2+ and Ca2+ metal ions. Moreover, it was stable on EDTA, Trixton-100, Tween 80, and Tween 20. Since* Bacillus subtilis* strain Btk27 was stable in most surfactants and inhibitors it could be applicable in various industries whenever pectin degradation is needed. The enzyme *K*m and *V*max values were identified as 1.879 mg/ml and 149.6 U, respectively. The potential application of the enzyme for coffee processing was studied, and it is found that complete removal of mucilage from coffee beans within 24 hours of treatment indicates the potential application in coffee processing.

## 1. Introduction

Biotechnological answers for environmental sustainability are modern solutions that help in the growth of the nation and are a boon for the welfare of human beings for the present and forthcoming generations. Biotechnology operations for enzyme production are no longer academic; it is a potentially useful alternative proposition for the future [1]. In this regard, pectinolytic enzymes can be applied in various industrial sectors wherever the degradation of pectin is required for a particular process. Several microorganisms have been used to produce different types of pectinolytic enzymes [2]. Microbial pectinases account for 25% of the global food and industrial enzyme sales [3, 4] and their market is increasing day by day. These are used extensively for fruit juice clarification, juice extraction, manufacture of pectin free starch, refinement of vegetable fibers, degumming of natural fibers, and wastewater treatment and as an analytical tool in the assessment of plant products [5, 6]. Pectinase treatment accelerates tea fermentation and also destroys the foam forming property of instant tea powders by destroying pectins. They are also used in coffee fermentation to remove mucilaginous coat from coffee beans [7, 8].

Parenthetically, Ethiopia is the original home of* Coffea arabica *L. and, thus, possesses the largest diversity in coffee genetic resources. Coffee is critical to the Ethiopian economy, since over 25% of the Ethiopia population depends on coffee for its livelihood. As per the past few years data, coffee production accounted on average for about 5% of Gross Domestic Product (GDP). Though Ethiopian exports continue to be dominated by basic commodities, share of coffee in total exports has shrunk from 53% to 31% during 2000–2012 [9].

Regardless of the importance of the crop, poor postharvest processing techniques largely contribute to the decline in coffee quality. The traditional processing practices employed by producers have imparted a negative impact on Ethiopian coffee quality. So far, few research attempts have been made to optimize with regard to fermentation for wet processing of coffee. Conventional coffee processing uses water to remove mucilage from coffee beans by natural fermentation. Quite often the mucilage breakdown is not complete even after 36–72 hour of fermentation. If the coffee beans are fermented for long hours, stinker beans (over fermented beans) develop. Most quality defects of coffee are attributed to incomplete mucilage removal and uncontrolled fermentation [10].

Previously, we screened microorganisms for the pectinase activity and identified* Bacillus subtilis strain* Btk 27 as potent pectinase producer. And we extensively studied the parameters for maximal pectinase production. The main aims of this study are to characterize the pectinase from* Bacillus subtilis strain* Btk 27 and testing the potential application in removal of mucilage from coffee beans.

## 2. Material and Methods

### 2.1. Inoculum Preparation

Fresh culture of* Bacillus subtilis strain* Btk 27 was inoculated into sterilized YEP medium with pH of 7.0 ± 0.5. The inoculated flask was incubated at 30°C on a rotary shaker at 120 rpm. Culture was grown in 50 ml media in 250 ml Erlenmeyer flasks.

### 2.2. Production of Pectinase

In 250 ml conical flask, 5.0 g of wheat bran was moistened by 75% of distilled water and autoclaved at 121° for 15 minute. The flasks were inoculated with 2.0 ml of overnight-grown seed culture of* Bacillus subtilis strain Btk 27*, mixed well to evenly distribute the inoculum, and incubated at 30°C for 48 h.

### 2.3. Extraction of Pectinase from Solid Substrate

Extraction of pectinase from ssf was done according to the method of Xiros et al. (2008) [11]. After 48 h of incubation 50 ml of distilled water was added into the solid substrate and the flasks are shaken for 1 h at 120 rpm on orbital shaker thoroughly and slurry is formed. Then, the flasks were kept at 4°C for 30 min under static conditions to facilitate the enzyme extraction. The slurry was centrifuged at 10,000*g* for 10 min at 4°C, and the clear supernatant was collected to assay the pectinase activity. The pectinase activity was determined in the supernatant as U/g of solid substrate used. The pectinase enzyme assay was based on the determination of reducing sugars produced as a result of enzymatic hydrolysis of pectin by dinitrosalicylic acid reagent (DNS) method (Miller, 1959). The enzyme unit was defined as the amount of enzyme that catalyzes *μ*mol of galacturonic acid per minute (*μ*mol min^−1^) under the assay conditions. Relative activity was calculated as the percentage of enzyme activity of the sample with respect to the sample for which maximum activity was obtained.

(1) Relative  Activity=Activity  of  sample U×100Maximum  enzyme  activity u.

### 2.4. Effect of Substrate Specificity on Pectinase Activity

The effect of substrate specificity on pectinase enzyme activity was determined by incubating 100 *µ*L of suitably diluted enzyme with 900 *µ*L of different substrates like Apple pectin, Citrus pectin, Xylan, and Galactose. These substrates were prepared in 0.1 M of phosphate buffer (pH 7.5) with 0.5% w/v concentration. The reaction mixture was incubated at 50°C for 10 minute and the enzyme activity assayed.

### 2.5. Effect of pH on Pectinase Activity

The effect of pH on pectinase activity was determined by incubating 900 *µ*L of substrate at different pHs with 100 *µ*L of suitably diluted enzyme at 50°C for 10 min and followed by assaying the enzyme activity. Substrate (0.5% w/v Citrus Pectin) was prepared at different pH values (pH 4.5–9.5) using different buffers (0.1 M) such as sodium acetate buffer, pH 4.5–6.0, phosphate buffer, pH 6.0–7.9, Tris-HCl buffer, pH 7.5–9.0, and glycine NaOH buffer, pH 8.5–10.0.

### 2.6. Effect of Temperature on Pectinase Activity

The effect of temperature on pectinase enzyme was evaluated by incubating the reaction mixture (900 *µ*L of substrate at different pHs with 100 *µ*L of suitably diluted enzyme) at different temperatures in the range of 30–80°C for 10 min with 5°C interval and the enzyme activity was assayed.

### 2.7. Effect of Surfactants and Inhibitors on Pectinase Activity

The effect of surfactants and inhibitors including mercaptoethanol, EDTA (1 mM), SDS (1%, w/v), Tween (20 and 80; 0.1%, v/v), and Triton X-100 (0.1%, v/v) on pectinase enzyme activity was studied by directly incorporating them into the enzyme substrate system. And then, the reaction mixture was incubated at 50°C for 10 min and the enzyme activity was assayed.

### 2.8. Effect of Metal Ions on Pectinase Activity

The effect of metal ions on pectinase activity was studied by directly incorporating them into the enzyme substrate system at a final concentration of 5 mM. Metal ions which were examined for their effect are Ca2+, Mg2+, Co2+, Cu2+, Fe3+, and Mn2+. The reaction mixture was incubated at 50°C for 10 min and the enzyme activity was assayed.

### 2.9. Thermostability of the Enzyme

The effect of enzyme stability under optimized temperature and optimized pH was studied by incubating the reaction mixture at various time intervals ranging 30, 60, 90, 120, 150, and 180 min.

### 2.10. Michaelis-Menten Constant (*K*m) and *V*max Values

The *K*m and *V*max values were determined by measuring the reaction velocity at different concentrations of the substrate (Citrus Pectin). First stock solution of Citrus Pectin which was of 10 mg/ml concentration was prepared with appropriate buffer (phosphate, pH 7.5). Then the stock solution was diluted by appropriate volume of buffer to make the final mg/ml Citrus Pectin concentrations listed in Table 1. The appropriate mg/ml Citrus Pectin (900 *µ*l) was incubated with 100 *µ*l of suitably diluted enzyme at 50°C for 10 minute and the pectinase enzyme activity was assayed.

The relationship between substrate (mg/ml of Citrus Pectin) and velocity (pectinase enzyme activity) was plotted using GraphPad Prism 5 software. The *K*m and *V*max values were calculated using nonlinear regression.

### 2.11. Removal of Mucilage from Coffee Beans Using Pectinase

Fresh coffee beans were harvested and pulped manually. The pulped beans were soaked with the enzyme mixture under static conditions until the mucilage was removed. Complete demucilisation was observed by hand feel as per traditional method; finally the demucilised coffee beans were washed and sun dried. To compare the enzymatic demucilisation with natural fermentation, the pulped coffee beans were soaked with water without enzyme addition.

## 3. Results

### 3.1. Effect of Substrate Specificity

The effect of substrate specificity on the activity of pectinase enzyme was determined by incubating the pectinase enzyme with different substrates (Table 2). The highest activity was observed when Citrus pectin was used as substrate. The effect of Citrus Pectin was significantly higher than the other tasted substrates.

### 3.2. Effect of pH

**Figure 1:**
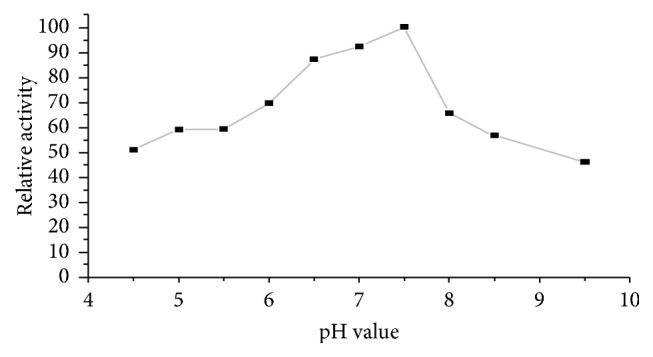
Effect of pH on activity of pectinase.

The effect of pH on pectinase activity was studied by incubating reaction mixture (Citrus Pectin and pectinase) at different pH values (pH 4.5–9.5). It was observed that the pectinase enzyme from* Bacillus subtilis* strain Btk 27 had highest activity at pH of 7.5 (Figure 1).

### 3.3. Effect of Temperature

**Figure 2:**
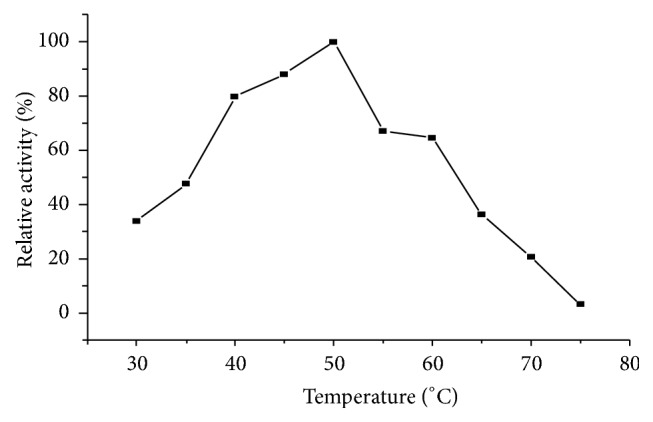
Effect of temperature on activity of pectinase.

The effect of temperature on pectinase enzyme was evaluated by incubating the reaction mixture at different temperatures in the range of 30–80°C. The maximum pectinase activity observed was at 50°C (Figure 2).

### 3.4. Effect of Inhibitors and Surfactants on Pectinase Activity

The effect of surfactants and inhibitors on pectinase activity was studied by directly incorporating them into the enzyme substrate system. Among the tasted surfactants and inhibitors, EDTA, Trixton-100, Tween 80, and Tween 20 enhanced the pectinase activity with relative activity of 165.3, 134.9, 100.4, and 153.6 (%), respectively. It was observed that the presence of Mercaptoethanol and SDS in the enzyme substrate system decreased pectinase activity significantly (Table 3).

### 3.5. Effect of Metal Ions

The effect of metal ions on pectinase activity was studied by directly incorporating them into the enzyme substrate system at a final concentration of 5 mM. The highest relative activities observed were 136.7% and 132.4% in the presence of Mg2+ and Ca2+ metal ions, respectively. The lowest activity observed was with the presence of Mn2+ metal ion (Table 4). However, the effect of these tested metal ions on pectinase activity was not significant.

### 3.6. Thermostability of the Enzyme

**Figure 3:**
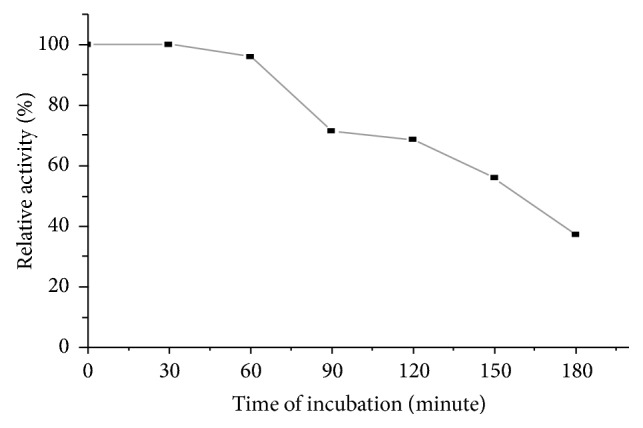
Enzyme stability.

The stability of pectinase enzyme under optimized temperature and pH was studied by incubating the reaction mixture at various time intervals (Figure 3). It was observed that the enzyme was stable with 100% relative activity until 60 minutes of incubation. However, beyond 60 minutes of incubation, the enzyme activity declined.

### 3.7. Michaelis-Menten Constant (*K*m) and *V*max Values

**Figure 4:**
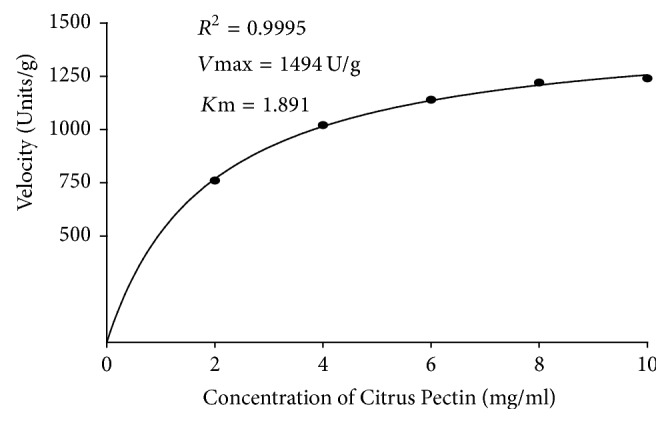
Michaelis-Menten Kinetics.

The *K*m and *V*max values of the enzyme were determined by measuring the reaction velocity at different concentrations of the substrate (Citrus Pectin). The relation between reaction velocity and the substrate concentration was analyzed with nonregression analysis. The regression coefficient (*R*^2^) was equal to 0.999 which describes the concentrations of Citrus Pectin and velocity (enzyme activity) readings were positively correlated (Figure 4). From the nonregression analysis, *K*m and *V*max values were identified as 1.891 mg/ml and 1494 U/g, respectively.

### 3.8. Potential Application of Pectinase on Demucilisation of Coffee Beans

**Figure 5:**
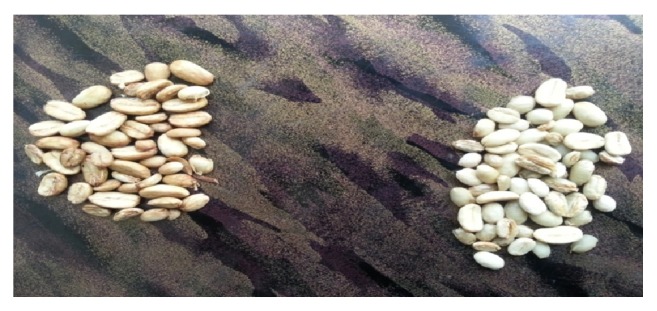
Removal of mucilage from coffee beans using natural fermentation (right) and using pectinase enzyme (left).

Fresh coffee beans were harvested and pulped manually. Half of the pulped beans were soaked into water that contained the crude pectinase whereas the other half were subjected to natural fermentation. Complete demucilisation was observed within 24 hours of incubation on pectinase treated coffee beans (Figure 5). However, in case of natural fermentation demucilisation was not completed even within 36 hour of fermentation.

## 4. Discussions

The maximum pectinase activity was observed when Citrus Pectin was used as substrate. Similarly, Celestino et al., (2006) [12] reported that novel pectinase enzyme from* Acrophialophora nainiana* showed the highest substrate activity on Citrus Pectin. Thus, it can be inferred that pectinase have high affinity for Citrus Pectin compared to others which are used in this study.

The optimum pH for pectinase activity was recorded at pH 7.5. Reports have shown pectinase activity to be highest around alkaline pH [13, 14]. Similar study on* B. stearothermophilus* showed optimum pectinase activities at pH 7.5 [15]. Moreover, pectinase from* Bacillus *sp. DT7 was maximally stable under alkaline conditions of pH 7.5–8.5 [16]. Therefore, this pectinase will have potential applications whenever alkaline pectin degradation is needed such as in coffee processing, paper and pulp industry, and Pectic waste water treatment.

The maximum pectinase activity was observed at 50°C; with further increase of temperature, the pectinase activity was decreased. This may be a result of thermal denaturation of the enzyme possibly due to disruption of noncovalent linkages, including hydrophobic interactions [17]. Likewise, Phutela et al. (2005) [18] reported an optimum temperature of 60°C for thermophiles* A. fumigates* pectinase. Alana et al., (1990) also reported that* Penicillium italicum *pectinase activity increase up to 50°C. The result might indicate that pectinase from* Bacillus subtilis* strain Btk27 is thermophilic enzyme.

Surfactant agent stability of the enzyme is one of the important parameters enabling enzymes to be used in different types of industries. In this study, the pectinase activity was stimulated on EDTA, Trixton-100, Tween-20, and Tween-80, whereas SDS significantly decreased pectinase activity. Moreover, Mercaptoethanol completely inhibited pectinase activity. Li et al., (2012) [19] reported that Tween-80 and Tween-20 stimulated the polygalacturonase activity. Zu-ming et al. (2008) [20] stated also surfactants such as Tween-80 and Tween-20 had stimulatory effects on pectinase activity. On the contrary, Amid et al. (2014) [21] reported that SDS, Trixton-100, and Tween-20 significantly reduced, Mercaptoethanol significantly increased, and EDTA had no significant effect on thermoalkaline pectinase. According to Zohdi and Amid, (2013) [22] most of the surfactants which interact with proteins cause distinct electrostatic and hydrophobic regions and alter the secondary or tertiary structure of enzymes. The stimulatory effect of some surfactants may be probably that the surfactants might improve the turnover number of pectinase by increasing the contact frequency between the active site of the enzyme and the substrate by lowering the surface tension of the aqueous medium [23]. Since Bacillus* subtilis* strain Btk27 was stable in most surfactants and inhibitors it could be applicable in various industries whenever pectin degradation is needed.

Among the metal ions, Mg2+, Zn2+, Co2+, and Fe2+ increased pectinase activity whereas Mn2+ decreased the pectinase activity; however their effect was not significant. Metal ions like Ca2+ and Mg2+ might play a vital role in maintaining the active confirmation of alkaline endo polygalacturonase to stimulate the activity [24]. Alana et al., (1990) [25] reported that Ca2+, Mg2+, Zn2+, and Mn2+ did not affect pectin lyase activity of* P. italicum* at 5 mM. This discrepancy in the divalent metal ion preference suggested that the enzymes might have differential flexibility in the active site. Beg and Gupta, (2003) [26] reported that metal ions such as Mg2+ and Ca2+ might play a vital role in maintaining the active confirmations of the alkaline pectinase to stimulate the activity.

Pectinase from* Bacillus subtilis* strain Btk27 was stable with 100% relative activity until 60 minutes of incubation. However, above 60 minutes of incubation the enzyme activity declined. Çelik et al. (2010) [27] reported that purified enzyme was stable and retained its full activity until 1 hour incubation period but the activity was reduced to 20% after 1 hour incubation. Gummadi and Panda (2003) [28] stated that the stability of pectinases is affected by both physical parameters (pH and temperature) and chemical parameters (inhibitors or activators). The thermal inactivation of enzymes is always due to denaturation of enzyme [29].

In enzymatic reaction, the kinetic parameter is also important, which describes enzyme efficiency. In this study, *V*max and *K*m values were 149.6 U and 1.88 mg/ml, respectively. Saad et al. (2007) [30] reported a *K*m of 1.88 mg/mL and *V*max of 0.045 mole/mL/min for* Mucor rouxii*. Celestino et al., (2006) [12] also reported that* Acrophialophora nainiana* had a *K*m value of 4.22 mg/ml. Moreover, Laha et al. (2014) [31] reported that* P. chrysogenum* had *K*m and *V*max values of 1.0 mg/mL and 78 U, respectively. Pectinase from Bacillus* subtilis* strain Btk27 relatively has the highest affinity for substrate due to its lowest *K*m; it also has the highest utility of pectin substrate as a result of its highest *V*max. As a result of this high binding of pectinase from* Bacillus subtilis* strain Btk27 with pectin substrate, small quantity of the enzyme will digest a considerably high amount of substrate. This may therefore reduce the cost for the enzyme in industrial use.

Pectinase are used in coffee processing to remove the mucilaginous coat from the coffee beans [32]. However, there is no reported application of pectinase in Ethiopia for processing coffee to date. In this study, pectinase was applied in small scale coffee processing, and complete removal of mucilage from coffee beans within 24 hours of incubation was observed. Murthy and Naidu (2011) [33] reported complete demusilisation of Robusta coffee within 36 hour of incubation. The enzyme treatment significantly reduces the fermentation time and holds up coffee quality loss due to traditional coffee processing.

## 5. Conclusion

The pectinase from* Bacillus subtilis* strain Btk27 was alkaline, thermophilic, and stable with many of tasted surfactants. In addition, It was observed that the pectinase from* Bacillus subtilis* strain Btk27 has huge promising potential in removal of mucilage from coffee beans.

## Figures and Tables

**Table 1:** mg/ml concentrations of Citrus Pectin to determine the *K*m and *V*max values.

Volume of stock solution (*µ*l)	Volume of buffer (*µ*l)	mg/ml of Citrus Pectin
180	720	2
360	540	4
540	360	6
720	180	8
900	0	10

**Table 2:** Effect of substrate specificity on pectinase activity.

Substrate	Enzyme activity (U/g)^*∗*^
Apple Pectin	441.53 ± 13.3^a^
Citrus Pectin	1272.4 ± 25.5^b^
Xylan	697.23 ± 11.73^c^
Galactose	0.0 ± 0.0^d^

(i) ^*∗*^Values are mean ± S.D. of 3 replicates; (a) values followed by different superscripts are significantly different at *P* < 0.05; (b) values followed by same superscripts are not significantly different at (*P* < 0.05).

**Table 3:** Effect of inhibitors and surfactants on pectinase activity.

Surfactant and Inhibitor	Enzyme activity (U/g)^*∗*^
Control	1272.4 ± 25.5^a^
EDTA	2103.3 ± 11.5^b^
Mercaptoethanol	0.0 ± 0.0^c^
SDS	697.6 ± 5.1^d^
Trixton-100	1715.9 ± 8.5^ab^
Tween 20	1954.4 ± 7.8^b^
Tween 80	1277.5 ± 11.1^a^

(i) ^*∗*^Values are mean ± S.D. of 3 replicates; (a) values followed by different superscripts are significantly different at *P* < 0.05; (b) values followed by same superscripts are not significantly different at (*P* < 0.05).

**Table 4:** Effects of metal ions on pectinase activity.

Metal Ion	Enzyme activity (U/g)^*∗*^
CaCl2	1684.6 ± 20.0^a^
CoCl2	1618.5 ± 9.3^a^
FeCl2	1528.2 ± 15.1^a^
MgCl2	1739.3 ± 31.8^a^
MnCl2	944.0 ± 38.7^a^
Control	1272.4 ± 25.5^a^

(i) ^*∗*^Values are mean ± S.D. of 3 replicates; (a) values followed by different superscripts are significantly different at *P* < 0.05; (b) values followed by same superscripts are not significantly different at (*P* < 0.05).
